# Breast arterial calcifications as a biomarker of cardiovascular risk: radiologists’ awareness, reporting, and action. A survey among the EUSOBI members

**DOI:** 10.1007/s00330-020-07136-6

**Published:** 2020-08-27

**Authors:** Rubina Manuela Trimboli, Davide Capra, Marina Codari, Andrea Cozzi, Giovanni Di Leo, Francesco Sardanelli

**Affiliations:** 1 Department of Biomedical Sciences for Health, Università degli Studi di Milano, Via Mangiagalli 31, Milan, 20133 Italy; 2 Department of Radiology, Stanford University School of Medicine, 300 Pasteur Dr, Stanford, CA 94305 USA; 3 Unit of Radiology, IRCCS Policlinico San Donato, Via Morandi 30, San Donato Milanese, 20097 Italy

**Keywords:** Cardiovascular diseases, Mammography, Mönckeberg medial calcific sclerosis, Survey and questionnaires, Radiologists

## Abstract

**Objectives:**

To investigate the knowledge of radiologists on breast arterial calcifications (BAC) and attitude about BAC reporting, communication to women, and subsequent action.

**Methods:**

An online survey was offered to EUSOBI members, with 17 questions focused on demographics, level of experience, clinical setting, awareness of BAC association with cardiovascular risk, mammographic reporting, modality of BAC assessment, and action habits. Descriptive statistics were used.

**Results:**

Among 1084 EUSOBI members, 378 (34.9%) responded to the survey, 361/378 (95.5%) radiologists, 263 females (69.6%), 112 males (29.6%), and 3 (0.8%) who did not specify their gender. Of 378 respondents, 305 (80.7%) declared to be aware of BAC meaning in terms of cardiovascular risk and 234 (61.9%) to routinely include BAC in mammogram reports, when detected. Excluding one inconsistent answer, simple annotation of BAC presence was declared by 151/233 (64.8%), distinction between low versus extensive BAC burden by 59/233 (25.3%), and usage of an ordinal scale by 22/233 (9.5%) and of a cardinal scale by 1/233 (0.4%). Among these 233 radiologists reporting BAC, 106 (45.5%) declared to orally inform the woman and, in case of severe BAC burden, 103 (44.2%) to investigate cardiovascular history, and 92 (39.5%) to refer the woman to a cardiologist.

**Conclusion:**

Among EUSOBI respondents, over 80% declared to be aware of BAC cardiovascular meaning and over 60% to include BAC in the report. Qualitative BAC assessment predominates. About 40% of respondents who report on BAC, in the case of severe BAC burden, investigate cardiovascular history and/or refer the woman to a cardiologist.

**Key Points:**

*• Of 1084 EUSOBI members, 378 (35%) participated: 81% of respondents are aware of breast arterial calcification (BAC) cardiovascular meaning and 62% include BAC in the mammogram report*.

*• Of those reporting BAC, description of presence was declared by 65%, low versus extensive burden distinction by 25%, usage of an ordinal scale by 10%, and of a cardinal scale by 0.4%; 46% inform the woman and, in case of severe BAC burden, 44% examine cardiovascular history, and 40% refer her to a cardiologist*.

*• European breast radiologists may be ready for large-scale studies to ascertain the role of BAC assessment in the comprehensive framework of female cardiovascular disease prevention*.

**Electronic supplementary material:**

The online version of this article (10.1007/s00330-020-07136-6) contains supplementary material, which is available to authorized users.

## Introduction

Cardiovascular disease still represents the leading cause of death for women [1]. Disparities in mortality rates still subsist by sex among age groups, with less favourable trends in young women [2, 3]. Recommended risk stratification algorithms—including traditional risk factors, sex, race, and ethnicity—do not adequately perform in women, leading to potential risk underestimation and subsequent undertreatment [4, 5]. Likely, traditional cardiovascular risk factors actually confer different risks for women and men. On the other hand, unique non-traditional risk factors such as pregnancy complications, oral contraception, hormonal fertility, menopausal therapies, and systemic autoimmune disorders play a crucial role in the complex biological pathway towards cardiovascular disease in women [6]. Moreover, the estrogenic dysregulation occurring in menopause—but also in premature ovarian insufficiency and obesity—favours breast arterial calcifications (BAC) development [7–10].

BAC on mammography are known to be strongly associated with cardiovascular disease, independently from other known cardiovascular risk factors [11], and their inclusion in cardiovascular risk algorithms has been advocated to improve cardiovascular outcomes in women [12, 13]. The current strength of this association, however, is not sufficient to warrant a clinical application in preventive cardiology. Indeed, the lack of validated and reproducible quantification methods allowing for risk stratification still represents an open issue. Nevertheless, no universal recommendations do exist on whether and how to report BAC and how to deal with them, discuss with women, or refer to cardiologists [5, 7].

In this context, the European Society of Breast Imaging (EUSOBI) launched a survey among its members to investigate the radiologist attitude about BAC awareness, reporting, communication, and actions. The results of this survey are reported in this paper.

## Materials and methods

### Survey design and recipients

The survey was developed by two board-certified radiologists, one (F.S.) with over 30 years of experience in breast and cardiovascular imaging and one (R.M.T.) with over 10 years of experience in breast imaging. A biomedical engineer with a background in image segmentation and survey methodology (M.C.) contributed to the design. After approval from the EUSOBI executive board, the questionnaire was published online on a dedicated software platform (Google Forms, Google). EUSOBI members were invited to anonymously participate by an e-mail from the society’s central office that included a link to the survey form. The self-administered questionnaire was available for 5 weeks, from February 10 to March 17, 2020, with two e-mail reminders sent on March 2 and March 15, 2020.

After a brief introduction about BAC and their association with cardiovascular risk, ten questions focused on the following: readers’ demographics (gender, age, geographical origin, job position) and experience (years in reading mammography, reading in a population-based screening mammography programme, number of mammograms read per year, percentage of working time dedicated to breast imaging); clinical setting (academic hospital, community hospital, private hospital, private practice). Subsequently, seven questions focused on whether participants had previously heard about the association between BAC and cardiovascular risk and whether they describe BAC in mammogram reports. If respondents indicated to include BAC in the mammogram reports, a further question investigated the method adopted for BAC assessment, distinguishing between qualitative and quantitative methods, according to whether visual subjective or numerical/objective evaluations were performed. Action habits, namely discussing with the woman her BAC status and, in case of severe BAC burden or progression, investigating cardiovascular anamnesis and/or referring to cardiologists, were also the focus of specific questions. The entire questionnaire is provided as Supplementary material (ESM 1).

### Statistical analysis

After survey closure on March 17, 2020, results were exported in a spreadsheet for statistical analysis. Descriptive statistics were expressed as absolute frequencies and percentages for categorical variables. To compare differences between variables, the chi-squared test was applied. Statistical analysis was performed using R v3.5.3 for Windows (The R Foundation for Statistical Computing).

## Results

### Demographics, experience, clinical setting, and job position

**Fig. 1:**
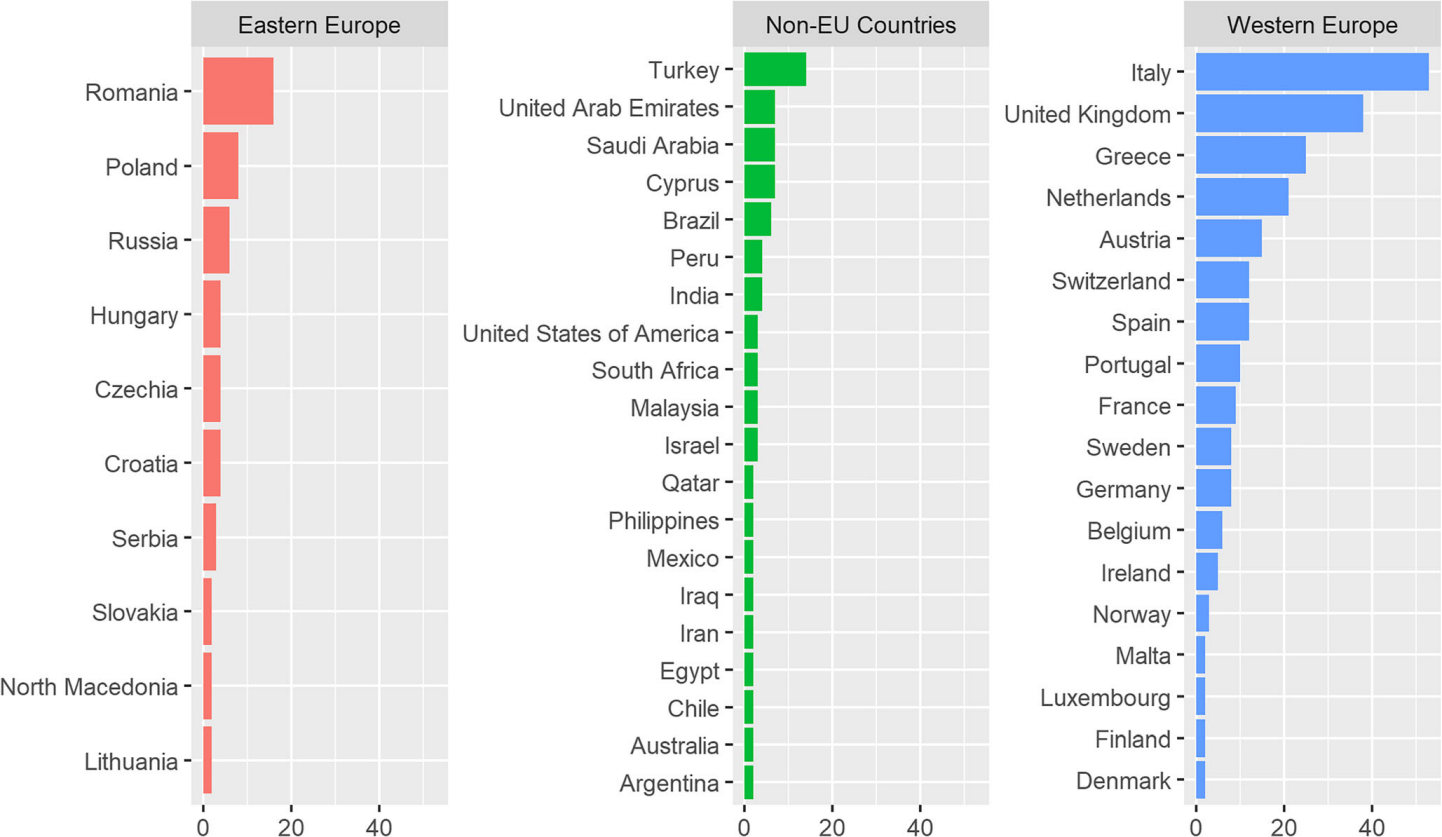
Geographic origin of participants to the survey, grouped by three different areas. Countries with less than two respondents were not plotted

Invitation e-mails were sent out to 1084 EUSOBI members, reaching 1074 of them. The questionnaire was filled-in by 378 participants, yielding a 35.2% response rate. Among them, 263 (69.6%) were females, 112 were males (29.6%), and 3 (0.8%) did not specify their gender. Most of the respondents were based in Europe (290/378, 76.7%), only 88/378 (23.2%) in non-European countries, mainly Turkey (14/378, 3.7%). Geographic origin was categorised in three different areas: Western (233/378, 61.6%) and Eastern Europe (57/378, 15.1%) and non-European countries (Fig. 1).

Board-certified radiologists mainly responded to the survey (361/378, 95.5%). Among them, 215 (59.6%) read in a population-based screening mammography programme and 87 (24.1%) are fully dedicated to breast imaging. Further characteristics of participants on demographics, experience, and clinical setting are given in Table 1.

**Table 1:** Participants' characteristics

	*n*	%
Demographics		
Gender		
Female	263	69.6
Male	112	29.6
Not specified	3	0.8
Age		
< 30 years	7	1.9
30–39 years	85	22.5
40–49 years	133	35.2
50–59 years	105	27.8
60–69 years	45	11.9
> 70 years	3	0.8
Job position		
Radiologist	361	95.5
Resident	8	2.1
Fellow/PhD	9	2.4
Clinical setting		
Academic hospital	152	40.2
Community hospital	83	22.0
Private hospital	75	19.8
Private practice	61	16.1
Other	7	1.9
Experience		
Reading mammography		
< 2 years	17	4.5
2–5 years	50	13.2
6–10 years	94	24.9
11–20 years	110	29.1
> 20 years	107	28.3
Screening reader		
Yes	224	59.3
No	154	40.7
Mammograms per year		
0–1999	132	34.9
2000–4999	127	33.6
> 5000	119	31.5
Working time in breast imaging		
0–99%	286	75.7
100%	92	24.3

### Awareness

Among the 378 respondents, 305 (80.7%) had heard about the association between BAC on mammography and cardiovascular risk. BAC awareness did not significantly differ among gender, geographic origin, clinical setting, job position, years of experience in reading mammography, number of mammograms read per year, and percentage of working time dedicated to breast imaging (*p* ≥ 0.101). Age and reading in a population-based screening mammography programme were associated with the knowledge of BAC cardiovascular meaning (*p* = 0.045 and *p* = 0.020, respectively). Respondents over 50 years were more frequently aware (131/153, 85.6%) compared with younger breast radiologists (174/225, 77.3%), while 76.8% (172/224) of those reading in a population-based screening mammography programme were aware of BAC meaning compared with 86.4% (133/154) of radiologists not involved in a population-based screening programme.

### Reporting

Over 60% of respondents (234/378, 61.9%) include BAC in the mammogram reports; 64.9% (198/305) of those aware of BAC cardiovascular meaning declared to report BAC compared with 49.3% (36/73) of those unaware (*p* = 0.014).

Attitude towards BAC reporting was not significantly influenced by gender, age, geographic origin, job position, years of experience in reading mammography, and percentage of working time dedicated to breast imaging (*p* ≥ 0.588). BAC reporting was associated to clinical setting and number of mammograms read per year (*p* < 0.001), as well as to reading in a population-based screening mammography programme (*p* = 0.037); 83.6% (51/61) of radiologists based in private practice and 66.7% (50/75) of those based in private hospitals use to report BAC, compared with 49.4% (41/83) of those based in community hospitals and 56.6% (86/152) of those based in academic hospitals; 68.3% (177/259) of respondents with a mammography workload under 5000 examinations per year and 68.2% (105/154) of those not reading in a population-based screening mammography programme declared to report BAC compared with 47.9% (57/119) of respondents having an annual mammography workload over 5000 examinations and 57.6% (129/224) of radiologist readers of population-based screening mammography. Association of BAC awareness and reporting with respondents’ demographics and experience are detailed in Table 2.

**Table 2:** Association of BAC awareness and reporting with respondents’ demographics and experience

	BAC awareness		*p*	BAC reporting		*p*
	Yes	No		Yes	No	
Demographics						
Gender			0.176			0.983
Female	206	57		163	100	
Male	96	16		69	43	
Not specified	3	0		2	1	
Age			0.045			0.588
< 50 years	174	51		142	83	
≥ 50 years	131	22		92	61	
Geographic origin			0.599			0.827
Western Europe	186	47		134	99	
Eastern Europe	47	10		39	18	
Non-EU countries	72	16		61	27	
Job position			0.139			0.718
Radiologist	294	67		225	136	
Resident	6	2		4	4	
Fellow/PhD	5	4		5	4	
Clinical setting			0.101			< 0.001
Academic hospital	129	23		86	66	
Community hospital	59	24		41	42	
Private hospital	59	16		50	25	
Private practice	52	9		51	10	
Other	6	1		6	1	
Experience						
Working time in breast imaging			0.401			0.796
0–99%	228	58		176	110	
100%	77	15		58	34	
Reading mammography			0.446			0.990
< 2 years	12	5		10	7	
2–5 years	39	11		30	20	
6–10 years	76	18		60	34	
11–20 years	86	24		68	42	
> 20 years	92	15		66	41	
Mammograms per year			0.360			< 0.001
0–1999	103	29		93	39	
2000–4999	101	26		84	43	
> 5000	101	18		57	62	
Screening reader			0.020			0.037
Yes	172	52		129	95	
No	133	21		105	49	
BAC awareness						0.014
Yes				198	107	
No				36	37	

### Assessment methods

**Fig. 2:**
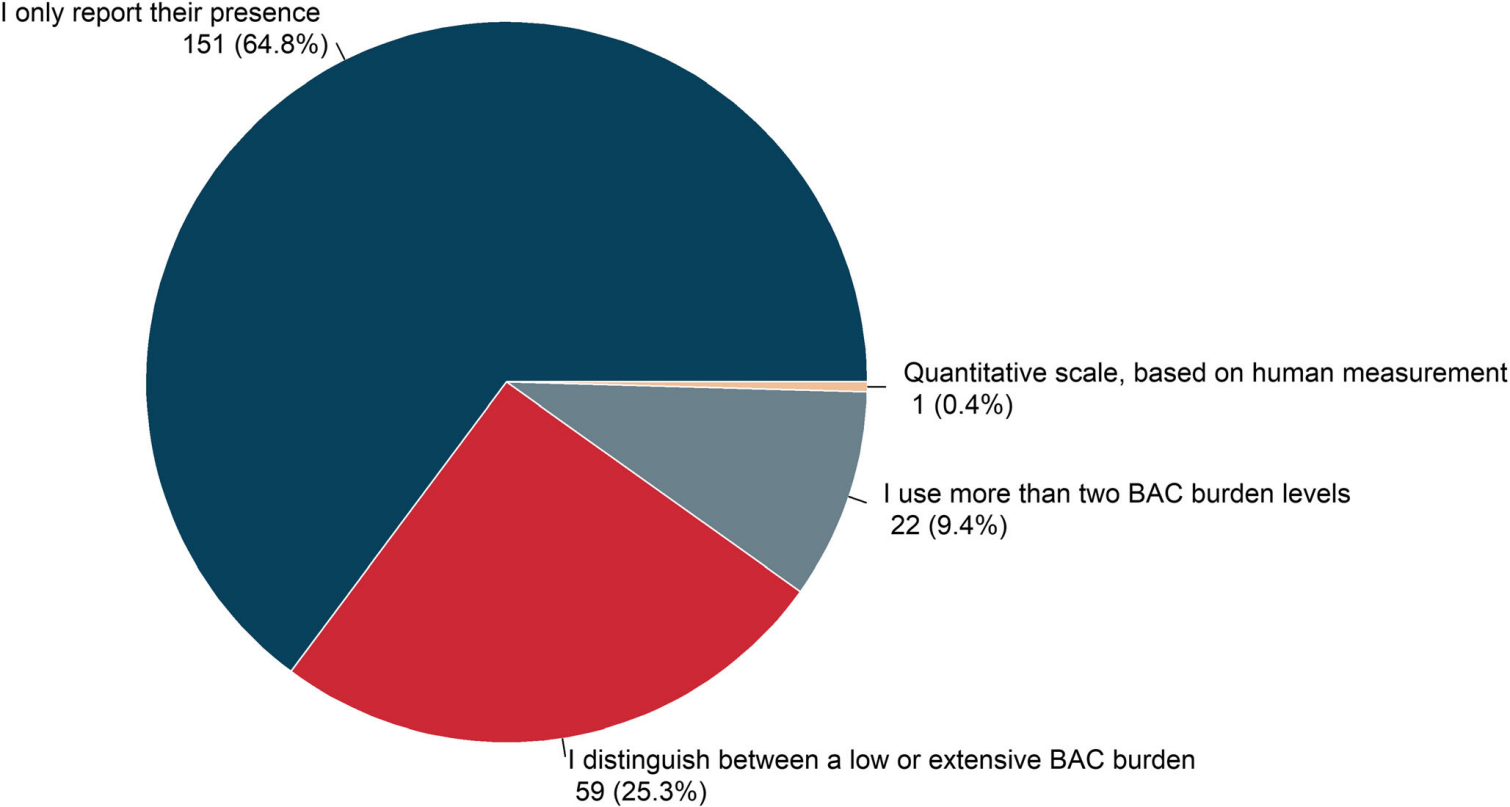
Pie chart of BAC assessment modality as declared by 233 BAC reporters. No one indicated computerised methods

Out of 234 responders who report BAC, one respondent gave an inconsistent answer that was excluded. Simple annotation of BAC presence was declared by 151/233 (64.8%), distinction between low versus extensive BAC burden by 59/233 (25.3%), usage of an ordinal scale by 22/233 (9.5%) and of a cardinal scale by 1/233 (0.4%). No respondent indicated the use of software for quantitative assessment (Fig. 2). The choice of the modality of assessment was not influenced by radiologists’ geographic origin, clinical setting, and experience (*p* ≥ 0.079). Gender and age were associated with the use of a dichotomous assessment method (*p* < 0.001), with female and younger than 50 years radiologists being more prone to adopt a dichotomic present/absent assessment, 73.5% (119/162) and 75.2% (106/141) respectively compared with 46.4% (32/69) males and 48.9% (45/92) radiologists over 50 years. Of note, 95/233 (40.8%) of respondents also evaluate BAC evolution over time by comparing previous examinations.

### Action habits

Three questions aimed to investigate radiologists’ attitude about communication of the BAC status to the woman and eventual subsequent actions to be taken. Among the 233 respondents reporting BAC, 106 (45.5%) orally inform the woman, 50 (47.2%) only in case of severe BAC burden or progression. Finally, in this case, 103/233 (44.2%) respondents investigate personal or family history of cardiovascular disease and 92/233 (39.5%) refer the woman to a cardiologist.

Overall, action habits were associated with respondents’ awareness about BAC, geographic origin, and clinical setting (*p* ≤ 0.048). Moreover, respondents under 50 years were less likely to communicate to the woman (*p* = 0.016) or to investigate cardiovascular anamnesis (*p* = 0.009). Action habits were not associated with radiologists’ experience (*p* ≥ 0.258). Significant associations of action habits with respondents’ demographics and experience are detailed in Table 3.

**Table 3:** Association of action habits with respondents’ demographics and BAC awareness

	Communication to the woman			*p*	Ask for CV anamnesis			*p*	Refer for a cardiology visit			*p*
	Yes	No	NR		Yes	No	NR		Yes	No	NR	
Demographics												
Age				0.016				0.009				0.191
< 50 years	55	85	2		53	88	2		51	89	2	
≥ 50 years	51	41	0		50	41	1		41	50	1	
Geographic origin				0.002				0.010				< 0.001
Western Europe	65	67	1		62	70	1		51	81	1	
Eastern Europe	24	15	0		23	16	0		26	12	1	
Non-EU countries	17	44	1		18	43	2		15	46	1	
Clinical setting				< 0.001				0.001				< 0.001
Academic hospital	28	58	0		34	52	1		26	59	1	
Community hospital	10	30	0		9	30	1		7	32	1	
Private hospital	32	17	1		28	22	1		25	25	1	
Private practice	36	15	0		31	20	0		31	20	0	
Other	0	6	0		1	5	0		3	3	0	
BAC awareness				0.002				0.029				0.048
Yes	98	98	1		93	103	2		83	112	2	
No	8	28	1		10	26	1		9	27	1	

*CV*, cardiovascular; *NR*, no response

## Discussion

The response rate to this survey, reaching more than a third of all EUSOBI members, shows a relatively high interest of breast radiologists towards BAC. Over 80% of participants were aware of the association between BAC and cardiovascular risk and over 60% declared to include BAC in mammography reports when they are present. Such data show how even in an era of highly specialised medicine and radiological subspecialties, about 30% of EUSOBI members display a comprehensive approach towards disease prevention, beginning to consider mammography as a tool that could combine breast cancer prevention with cardiovascular prevention in women.

There is robust evidence linking BAC with well-known cardiovascular risk factors, such as increasing age, parity, diabetes mellitus, and hyperlipidaemia, all associated with a higher BAC prevalence [5, 7, 11] than that (12.7%) currently reported among the general female population attending breast cancer screening programmes [11]. Several studies suggested that BAC presence is associated with a risk of coronary heart disease up to three times higher than in the general population [8, 14–16], independently from other known cardiovascular risk factors and from the presence of coronary artery disease [14, 15].

Notably, BAC and coronary atherosclerotic plaques are the expression of two different pathophysiological processes. While BAC are the consequence of Mönckeberg medial calcific sclerosis—a non-occlusive disease resulting in thicker and stiffer vessels—coronary plaques involve the vascular intima, leading to luminal narrowing and vessel occlusion [17].

This survey also revealed that, among radiologists reading in a population-based screening mammography programme, the majority is aware of BAC cardiovascular meaning and includes BAC in mammogram reports (when BAC are present), even if report forms, per se, do not provide such input. This is a positive proactive behaviour. First of all, this improves primary care physicians’ perception of BAC meaning and potential. Secondly, it facilitates the creation of large datasets, encouraging prospective studies with known cardiovascular outcomes to ascertain if BAC may improve cardiovascular risk stratification over traditional approaches. Finally, from the patient’s perspective, receiving such information is desirable. A recent study centred on a self-administered survey involving 419 women undergoing screening or diagnostic mammography highlighted how women have an overwhelming preference to be informed if BAC are found in their mammograms [18]. Of note, while cardiovascular risk awareness among women remains low despite the high toll paid in terms of cardiovascular mortality [19, 20], findings by Margolies et al [18] strongly hint that, if properly informed, women would become actively involved in tailored preventive strategies focused on cardiovascular disease, beginning to consider it not only as a “male problem”.

Oral communication with women about their BAC status is pursued by 45.5% of respondents which routinely include BAC in mammography reports, even though in 47.2% of cases doing it only when BAC burden or progression is deemed severe. If so, 44.2% proceed to investigate cardiovascular anamnesis and 39.5% refer women to a cardiologist. Interestingly, all these action habits were more frequently undertaken in Eastern European countries and by radiologists working in private hospitals and private practice. Such findings may be partially explained considering that these three geographic areas (Western Europe, Eastern Europe, and non-European countries) do not mirror official indications, rather reflect a similarity of routine practices both in screening and diagnostic setting.

Italy, along with the UK, had the greater number of respondents to this survey (53/378, 14.0% Italian and 38/378, 10.1% English respondents), as for the greater number of members within EUSOBI (187/1084, 17.2% Italian and 80/1084, 7.4% English members). This result could also be influenced by the lesser diffusion of organised screening programmes in some countries, as well as by differences in the number of examinations and allotted time performed in private setting, factors which unfortunately do not emerge from our survey. Finally, different socio-cultural contexts or medico-legal issues may also come into play.

Most of the respondents, 64.8%, qualitatively describe BAC as “present”, while just over a fourth assess them through an ordinal visual scale, the latter undoubtedly representing a considerable step from a mere subjective and qualitative evaluation towards a semi-quantitative assessment. Only one radiologist (0.4%) declared to manually perform a quantitative assessment, while computer-aided tools were absolutely missing.

Binary BAC classifications hinder the comprehensive stratification of women into multiple cardiovascular risk levels and the identifications of women who would mostly benefit from a tailored disease prevention [7]. The target population for a cost-effective further risk assessment is primarily represented by individuals at intermediate cardiovascular risk; indeed, in a low-risk population, the rationale for preventive intervention is missing, while in a high-risk population, further risk assessment would not reasonably impact on a formerly recommended pharmacological treatment [21]. Nevertheless, the major source of bias for qualitative or semi-quantitative methods for BAC evaluation has been—up to now—their poor reproducibility [7, 22]. Albeit easily detectable on mammography, BAC topological complexity and vessel overlap on two-dimensional mammographic projections make both the identification and quantification of BAC quite difficult to standardise, preventing robust validation and subsequent clinical application [5, 7]. A sound and reproducible BAC quantification, ideally through a continuous scale, is paramount for cardiovascular risk stratification. Valuable information on this issue will probably come from the MINERVA study, the first large prospective study using a continuous mass (in milligrams) score for BAC assessment. These results will hopefully contribute to determine whether this validated method [23] is indeed capable of predicting coronary heart disease, cerebrovascular disease, heart failure, peripheral vascular disease, and total cardiovascular disease on a large population. Answers to these questions will also clarify if adding BAC burden classification to prediction models based on traditional risk factors effectively stimulates a reclassification of cardiovascular disease risk in women [24].

An automated tool could also represent a useful solution for BAC quantification and is what we must strive for. Recently, the use of artificial intelligence systems for BAC segmentation was investigated. In particular, a deep convolutional neural network was trained to discriminate between BAC and non-BAC pixels from digital mammography images, obtaining good performances both in BAC detection and calcium mass quantification [25]. However, such systems, although promising, are still far from being validated and subsequently applied, large-scale studies being needed to obtain further improvements.

Limitations of this survey include, first, a potential selection bias among radiologist members of the EUSOBI, who of course supposedly display special interest in breast imaging and in keeping updated with correlated research on such topic. Therefore, awareness about BAC meaning and reporting attitude are potentially overestimated and not generalisable to the whole radiological community. Secondly, the response rate was below 50%, as indeed typically observed in similar surveys [26], with our 35.2% response rate being higher than expected and representing an excellent data. Finally, more detailed questions about education, habits, and technical challenges were avoided to reach a good compliance in terms of response rate.

In conclusion, this survey illustrated that, among EUSOBI members, more than 80% of respondents are aware of BAC implications in terms of cardiovascular risk, and that more than 60% include BAC on mammogram reports when they are present. However, BAC quantitative estimates are not performed, with very few exceptions.

Large-scale studies are now needed to ascertain the role of BAC assessment in the comprehensive framework of female cardiovascular disease prevention, provided that BAC quantitative methods are available. Furthermore, efforts should be pursued in discussing with women their BAC status and its meaning. Reporting BAC may prove a step towards promoting cardiovascular disease prevention in women via mammography. This would ultimately confer the ability to concomitantly influence the course of two leading causes of death in women, i.e. breast cancer and cardiovascular disease, to a widely diffused and endorsed population-based screening programme.

## Electronic supplementary material

ESM 1 (PDF 177 kb)

## Abbreviations

BAC: Breast arterial calcifications
EUSOBI: European Society of Breast Imaging

## References

[1] GBD 2017 Causes of Death Collaborators (2018) Global, regional, and national age-sex-specific mortality for 282 causes of death in 195 countries and territories, 1980–2017: a systematic analysis for the Global Burden of Disease Study 2017. Lancet 392:1736–1788 10.1016/S0140-6736(18)32203-7 PMC6227606 30496103

[2] Benjamin EJ, Muntner P, Alonso A (2019). Heart disease and stroke statistics—2019 update: a report from the American Heart Association. Circulation.

[3] Wilmot KA, O'Flaherty M, Capewell S, Ford ES, Vaccarino V (2015) Coronary heart disease mortality declines in the United States from 1979 through 2011. Circulation 132:997–1002 10.1161/CIRCULATIONAHA.115.015293 PMC4828724 26302759

[4] DeFilippis AP, Young R, Carrubba CJ (2015). An analysis of calibration and discrimination among multiple cardiovascular risk scores in a modern multiethnic cohort. Ann Intern Med.

[5] Bui QM, Daniels LB (2019). A review of the role of breast arterial calcification for cardiovascular risk stratification in women. Circulation.

[6] Wenger NK (2015). Transforming cardiovascular disease prevention in women: time for the pygmalion construct to end. Cardiology.

[7] Trimboli RM, Codari M, Guazzi M, Sardanelli F (2019). Screening mammography beyond breast cancer: breast arterial calcifications as a sex-specific biomarker of cardiovascular risk. Eur J Radiol.

[8] Schnatz PF, Marakovits KA, OʼSullivan DM (2011). The association of breast arterial calcification and coronary heart disease. Obstet Gynecol.

[9] Schnatz PF, Rotter MA, Hadley S, Currier AA, O'Sullivan DM (2007) Hormonal therapy is associated with a lower prevalence of breast arterial calcification on mammography. Maturitas 57:154–160 10.1016/j.maturitas.2006.12.002 17289309

[10] Anagnostis P, Paschou SA, Katsiki N, Krikidis D, Lambrinoudaki I, Goulis DG (2019) Menopausal hormone therapy and cardiovascular risk: where are we now? Curr Vasc Pharmacol 17:564–572 10.2174/1570161116666180709095348 29984659

[11] Hendriks EJE, De Jong PA, van der Graaf Y, Mali WPTH, van der Schouw YT, Beulens JWJ (2015) Breast arterial calcifications: a systematic review and meta-analysis of their determinants and their association with cardiovascular events. Atherosclerosis 239:11–20 10.1016/j.atherosclerosis.2014.12.035 25568948

[12] Iribarren C, Molloi S (2013). Breast arterial calcification: a new marker of cardiovascular risk?. Curr Cardiovasc Risk Rep.

[13] Abouzeid C, Bhatt D, Amin N (2018). The top five women’s health issues in preventive cardiology. Curr Cardiovasc Risk Rep.

[14] van Noord PA, Beijerinck D, Kemmeren JM, van der Graaf Y (1996). Mammograms may convey more than breast cancer risk: breast arterial calcification and arterio-sclerotic related diseases in women of the DOM cohort. Eur J Cancer Prev.

[15] Kemmeren JM, van Noord PAH, Beijerinck D, Fracheboud J, Banga JD, van der Graaf Y (1998) Arterial calcification found on breast cancer screening mammograms and cardiovascular mortality in women: the DOM project. Am J Epidemiol 147:333–341 10.1093/oxfordjournals.aje.a009455 9508100

[16] Iribarren C, Go AS, Tolstykh I, Sidney S, Johnston SC, Spring DB (2004) Breast vascular calcification and risk of coronary heart disease, stroke, and heart failure. J Womens Health (Larchmt) 13:381–389 10.1089/154099904323087060 15186654

[17] Zazzeroni L, Faggioli G, Pasquinelli G (2018). Mechanisms of arterial calcification: the role of matrix vesicles. Eur J Vasc Endovasc Surg.

[18] Margolies LR, Yip R, Hwang E et al (2019) Breast arterial calcification in the mammogram report: the patient perspective. AJR Am J Roentgenol 212:209–214 10.2214/AJR.18.20171 30354267

[19] Wenger N (2002). Clinical characteristics of coronary heart disease in women: emphasis on gender differences. Cardiovasc Res.

[20] Bairey Merz CN, Andersen H, Sprague E (2017). Knowledge, attitudes, and beliefs regarding cardiovascular disease in women. J Am Coll Cardiol.

[21] Degrell P, Sorbets E, Feldman LJ, Steg PG, Ducrocq G (2015) Screening for coronary artery disease in asymptomatic individuals: why and how? Arch Cardiovasc Dis 108:675–682 10.1016/j.acvd.2015.10.001 26596251

[22] Trimboli RM, Codari M, Bert A (2018). Breast arterial calcifications on mammography: intra- and inter-observer reproducibility of a semi-automatic quantification tool. Radiol Med.

[23] Molloi S, Mehraien T, Iribarren C, Smith C, Ducote JL, Feig SA (2009) Reproducibility of breast arterial calcium mass quantification using digital mammography. Acad Radiol 16:275–282 10.1016/j.acra.2008.08.011 PMC2664654 19201356

[24] Iribarren C, Sanchez G, Husson G (2018). MultIethNic study of brEast aRterial calcium gradation and cardioVAscular disease: cohort recruitment and baseline characteristics. Ann Epidemiol.

[25] Wang J, Ding H, Bidgoli FA (2017). Detecting cardiovascular disease from mammograms with deep learning. IEEE Trans Med Imaging.

[26] Clauser P, Mann R, Athanasiou A (2018). A survey by the European Society of Breast Imaging on the utilisation of breast MRI in clinical practice. Eur Radiol.

