# Supplementary material for: Breast arterial calcifications as a biomarker of cardiovascular risk: radiologists’ awareness, reporting, and action. A survey among the EUSOBI members
Source: Eur Radiol. 2020 Aug 27;31(2):958–66. doi: 10.1007/s00330-020-07136-6 (PMC7813731; doi:10.1007/s00330-020-07136-6)
Supplement: Supplementary file 1 — (PDF 177 kb) [file 330_2020_7136_MOESM1_ESM.pdf]

Breast arterial calcifications (BAC) reporting survey

*Required

1. Are you:

Mark only one oval.

- Female
- Male
- Prefer not to answer

2. Your age is: *

Mark only one oval.

- <30 years
- 30-39 years
- 40-49 years
- 50-59 years
- 60-69 years
- >70 years

3. In what country do you work? *

4. Are you based in: *

Mark only one oval.

- Academic Hospital
- Community Hospital
- Private Hospital
- Private Practice
- Other

5. What is your current position? *

Mark only one oval.

- Radiologist
- Fellow\PhD
- Resident
- Other

6. How many years of experience do you have in breast imaging? *

Mark only one oval.

- <2 years
- 2-5 years
- 6-10 years
- 11-20 years
- >20 years

7. How many years of experience do you have in reading mammography? *

Mark only one oval.

- <2 years
- 2-5 years
- 6-10 years
- 11-20 years
- >20 years

8. Are you a population-based screening reader? *

Mark only one oval.

- Yes
- No

9. How many mammograms do you read per year? *

Mark only one oval.

- <500
- 500-999
- 1000-1999
- 2000-2999
- 3000-3999
- 4000-4999
- >5000

10. What percentage of your time is dedicated to breast imaging? *

Mark only one oval.

- 100%
- 80-99%
- 60-79%
- 40-59%
- 20-39%
- <20%

11. Have you heard about the association between BAC on mammography and cardiovascular risk? *

Mark only one oval.

- Yes
- No

12. Do you report on BAC in diagnostic mammography reports? *

Mark only one oval.

- Yes
- No

13. How do you report BAC?

Mark only one oval.

- I ignore BAC
- I only report their presence
- I distinguish between a low or extensive BAC burden
- I use more than two BAC burden levels (e.g. minimal, mild, moderate, severe)
- Quantitative scale, based on human measurement
- Computer-based quantitative scale

14. Do you assess the progression of BAC comparing current to previous mammograms?

Mark only one oval.

- Yes
- No

15. If you see BAC, do you orally inform the woman?

Mark only one oval.

- Yes
- No
- Only if I evaluate the BAC burden/progession as severe

16. If you see severe BAC burden or progression, do you ask the woman for personal and family history of cardiovascular disease/events?

Mark only one oval.

- Yes
- No

17. If you see severe BAC burden or progression in comparison to previous mammograms, do you refer the woman for a cardiology visit?

Mark only one oval.

- Yes
- No

18. Any suggestions or comments (general or referred to specific questions)?

This content is neither created nor endorsed by Google.

Google Forms
